# Supplementary material for: Robust Moiety Model Selection Using Mass Spectrometry Measured Isotopologues
Source: Metabolites. 2020 Mar 21;10(3):118. doi: 10.3390/metabo10030118 (PMC7143054; doi:10.3390/metabo10030118)
Supplement: Supplementary file 1 [file metabolites-10-00118-s001.pdf]

Supporting Information for

Robust Moiety Model Selection Using Mass Spectrometry Measured Isotopologues

Huan Jin [1] and Hunter N.B. Moseley[2, 3, 4, 5, *]

[1] Department of Toxicology and Cancer Biology; huan.jin@uky.edu
[2] Department of Molecular & Cellular Biochemistry
[3] Markey Cancer Center
[4] Resource Center for Stable Isotope Resolved Metabolomics
[5] Institute for Biomedical Informatics

University of Kentucky, Lexington KY, United States

* Correspondence: hunter.moseley@uky.edu; Tel.: 859-218-2964

Table S1. Comparison of mode rank based on different model selection criteria.

Models	AICc	rank	AIC	rank	BIC	rank
**Expert-derived model**	**-401.7597**	**1**	**-421.3026**	**1**	**-385.5009**	**1**
7_G1R1A2U3	-384.3075	2	-413.1825	2	-371.4139	2
7_G2R1A1U3_g5	-381.2868	3	-410.1618	3	-368.3932	3
7_G1R2A1U3_r3	-379.2657	4	-408.1407	4	-366.3720	4
7_G1R2A1U3_r4	-378.8969	5	-407.7719	5	-366.0033	5
7_G2R1A1U3_g4	-375.9538	6	-404.8288	6	-363.0601	6
6_G1R1A1U3_g5	-374.9694	7	-394.5122	10	-358.7105	8
6_G1R1A1U3_r4	-374.1820	8	-393.7249	11	-357.9231	9
7_G1R1A1U4	-373.4563	9	-402.3313	7	-360.5626	7
6_G1R1A1U3_u4	-370.0716	10	-389.6145	13	-353.8127	11
7_G2R1A1U3_g1	-367.8353	11	-396.7103	8	-354.9416	10
7_G2R1A1U3_g2	-360.1668	12	-389.0418	14	-347.2732	13
7_G1R1A1U3C1	-360.0296	13	-388.9046	15	-347.1360	14
7_G1R2A1U3_r1	-354.8814	14	-383.7564	16	-341.9878	16
8_G1R2A2U3_r3	-354.4480	15	-395.8273	9	-348.0917	12
8_G2R1A2U3_g4	-351.9886	16	-393.3679	12	-345.6323	15
6_G0R2A1U3_g3r2r3_g6r5	-345.1277	17	-364.6706	21	-328.8689	17
8_G2R1A2U3_g1	-334.2882	18	-375.6675	17	-327.9319	18
7_G2R1A1U3_g3	-332.9148	19	-361.7898	22	-320.0211	19
7_G1R2A1U3_r2	-332.3262	20	-361.2012	23	-319.4326	21
8_G1R2A2U3_r1	-325.9344	21	-367.3137	18	-319.5781	20
8_G1R1A2U3C1	-324.5196	22	-365.8989	19	-318.1633	22
8_G2R1A2U3_g5	-324.5004	23	-365.8797	20	-318.1441	23
7_G0R2A2U3_g3r2r3_g6r5	-324.0749	24	-352.9499	26	-311.1813	25
7_G1R2A1U3_g3r2r3	-324.0721	25	-352.9471	27	-311.1784	26
8_G2R1A2U3_g2	-318.5771	26	-359.9564	24	-312.2208	24
6_G1R1A1U3_a1	-318.2498	27	-337.7927	31	-301.9910	28
8_G1R2A2U3_r4	-317.3169	28	-358.6962	25	-310.9606	27
8_G2R1A2U3_g3	-302.7897	29	-344.1690	28	-296.4334	29
8_G1R2A2U3_g3r2r3_g6r5_g5	-297.7429	30	-339.1222	30	-291.3866	30
8_G1R2A2U3_r2r3	-295.0078	31	-336.3871	32	-288.6515	31
8_G1R2A2U3_r2	-294.7900	32	-336.1693	33	-288.4337	32
8_G1R2A2U3_g3r2r3	-292.7867	33	-334.1660	34	-286.4304	33
9_G2R2A2U3_r2r3_g6r5_g3_g5	-281.8920	34	-340.0458	29	-286.3433	34
7_G0R3A1U3_g3r2r3_g6r5_g5r4	-279.0349	35	-307.9099	37	-266.1412	36
9_G2R2A2U3_r2r3_g4	-273.5807	36	-331.7345	35	-278.0320	35
9_G2R2A2U3_r2r3_g5	-254.4087	37	-312.5625	36	-258.8599	37
9_G2R2A2U3_r2r3_g3	-248.2277	38	-306.3815	38	-252.6789	38
9_G2R2A2U3_r2r3_g2	-242.9984	39	-301.1522	39	-247.4497	39
9_G2R2A2U3_r2r3_g1	-242.4110	40	-300.5648	40	-246.8623	40
7_G0R3A1U3_g3r2r3_g6r5_r4	-226.7271	41	-255.6021	41	-213.8334	41

Dataset: FT-ICR-MS (combined); Optimization method: SAGA-optimize (25000 steps); Objective function: Absolute difference.

Table S2. Model selection test with absolute difference objective function.

Optimization steps	Loss value	AICc	Selected model
500	0.840	-344.734	Expert-derived model
1000	0.682	-368.696	Expert-derived model
2000	0.580	-386.000	Expert-derived model
5000	0.492	-398.243	Expert-derived model
10000	0.447	-402.611	Expert-derived model
15000	0.430	-405.722	Expert-derived model
25000	0.458	-407.414	6_G1R1A1U3

Dataset: LC-MS (split); Selection criterion: AICc

Table S3. Model selection test with square difference objective function.

Optimization steps	Loss value	AICc	Selected model
500	0.031	-348.250	Expert-derived model
1000	0.021	-368.818	Expert-derived model
2000	0.015	-387.196	Expert-derived model
5000	0.011	-404.563	Expert-derived model
10000	0.010	-411.177	Expert-derived model
15000	0.010	-413.499	Expert-derived model
25000	0.009	-415.498	Expert-derived model

Dataset: LC-MS (split); Selection criterion: AICc

Table S4. Model selection test with absolute difference of logs objective function.

Optimization steps	Loss value	AICc	Selected model
500	50.595	-315.616	Expert-derived model
1000	47.213	-319.026	Expert-derived model
2000	44.137	-323.619	Expert-derived model
5000	40.811	-320.949	Expert-derived model
10000	39.474	-328.551	Expert-derived model
15000	57.856	-331.700	6_G1R1A1U3

Dataset: LC-MS (split); Selection criterion: AICc

Table S5. Model selection test with difference of AIC objective function.

Optimization steps	Loss value	Selected model
500	-365.957	Expert-derived model
1000	-389.987	Expert-derived model
2000	-409.322	Expert-derived model
5000	-427.064	Expert-derived model
10000	-435.618	Expert-derived model
15000	-437.970	Expert-derived model
25000	-439.533	Expert-derived model

Dataset: LC-MS (split); Selection criterion: AICc

Table S6. Inclusion of less informative dataset can lead to failure in model selection.

Optimization step	Selected model				
	**5 time points (0-36h)**	**4 time points (6-36h)**	**3 time points (12-36h)**	**2 time points (24-36h)**	**1 time point (36h)**
500	ED model	ED model	ED model	ED model	ED model
1000	ED model	ED model	ED model	ED model	ED model
2000	ED model	ED model	ED model	ED model	ED model
5000	6_G1R1A1U3_u4	6_G1R1A1U3_u4	ED model	ED model	7_G1R2A1U3_r1
10000	6_G1R1A1U3_u4	6_G1R1A1U3_u4	ED model	7_G1R2A1U3_r1	7_G1R2A1U3_r1
15000	6_G1R1A1U3_u4	6_G1R1A1U3_u4	6_G1R1A1U3_u4	7_G1R2A1U3_r1	7_G1R2A1U3_r1
25000	6_G1R1A1U3_u4	6_G1R1A1U3_u4	6_G1R1A1U3_u4	7_G1R2A1U3_r1	7_G1R2A1U3_r1

Dataset: LC-MS (split); Objective function: log difference; Selection criterion: AICc; Optimization method: SAGA-optimize.

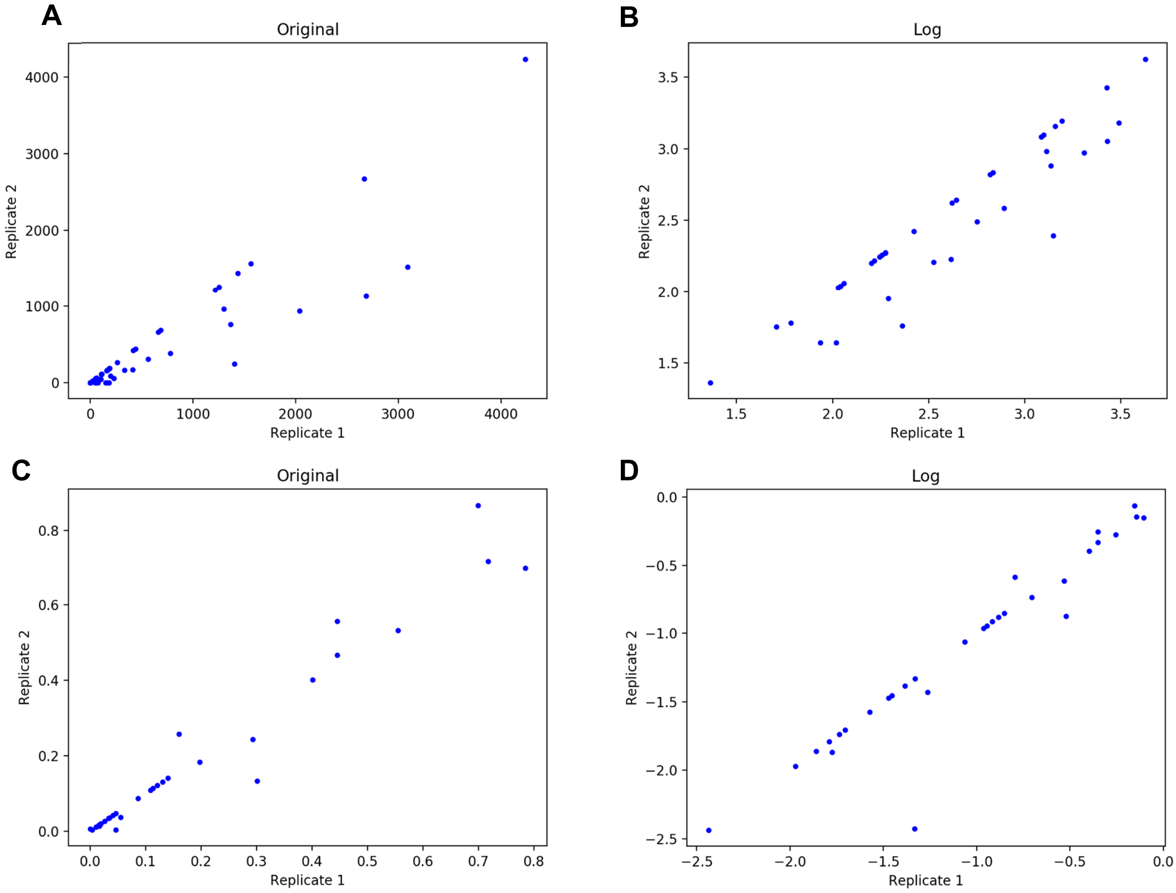

Figure S1. Error analysis in FT-ICR-MS datasets. A and B are plots of raw data. C and D are plots of renormalized data after natural abundance correction. All these plots contain all time points.

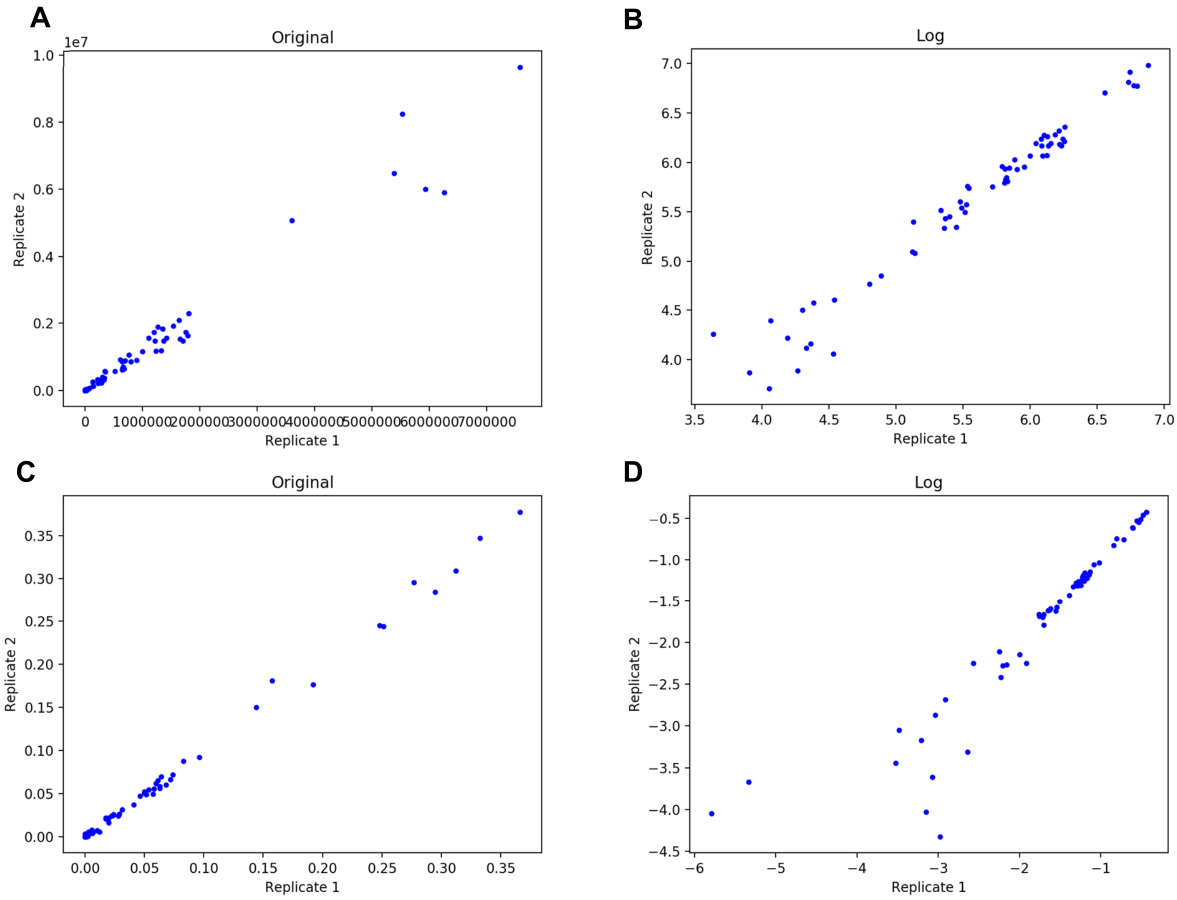

Figure S2. Error analysis in LC-MS datasets. A and B are plots of raw data. C and D are plots of renormalized data after natural abundance correction. All these plots contain all time points.

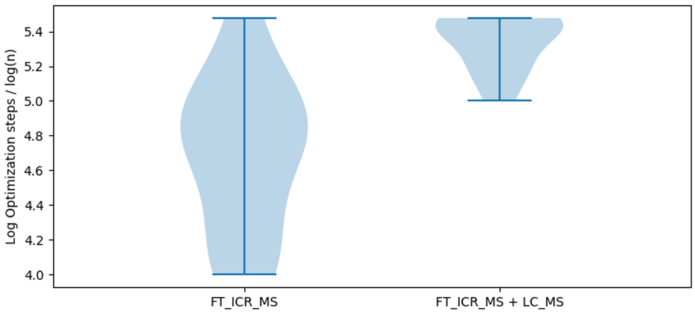

Figure S3. Comparison of the log of optimization steps where model selection with different datasets begins to fail with absolute difference of logs objective function.
